# Metabolic engineering of *Yarrowia lipolytica* for enhanced microbial production of medium-chain α, ω-diols from alkanes via CRISPR-Cas9 mediated pathway optimization and P450 alkane monooxygenase overexpression

**DOI:** 10.3389/fbioe.2025.1695661

**Published:** 2025-10-23

**Authors:** Ye Chan Kim, Won Yoon Hwan Choi, Eun-Jung Kim, Byung‐Gee Kim, Hyungdon Yun

**Affiliations:** ^1^ Department of Systems Biotechnology, Konkuk University, Seoul, Republic of Korea; ^2^ EGC Therapeutics, Inc., Seoul, Republic of Korea; ^3^ School of Chemical and Biological Engineering, Seoul National University, Seoul, Republic of Korea

**Keywords:** metabolic engineering, CRISPR-Cas9, diols, alkanes, hydroxylation, yarrowia lipolytica

## Abstract

**Introduction:**

Medium- to long-chain α, ω-diols are valuable building blocks for polyesters and polyurethanes, yet their microbial synthesis from inexpensive alkane feedstocks remains largely undeveloped. The oleaginous yeast Yarrowia lipolytica offers advantages over bacterial systems such as Escherichia coli for alkane conversion due to its inherent capacity to metabolize hydrophobic substrates.

**Methods:**

To enable de novo α, ω-diol production from alkanes, we used CRISPR-Cas9 to delete ten genes involved in fatty alcohol oxidation (including FADH, ADH1 -8, and FAO1) and four genes linked to fatty aldehyde oxidation (FALDH1 -4). This generated the strain YALI17, with reduced over-oxidation activity. Further metabolic enhancement was achieved by overexpressing alkane hydroxylase genes, particularly ALK1. Fermentation performance was evaluated under controlled pH conditions using n-dodecane as the substrate.

**Results:**

The engineered strain YALI17 produced 1,12-dodecanediol at 0.72 mM from 50 mM n-dodecane –a 14-fold increase relative to the parental strain. ALK1 overexpression in YALI17 further raised production to 1.45 mM, and automated pH-controlled biotransformation achieved 3.2 mM.

**Discussion:**

This study demonstrates the first successful biotransformation of medium- to long-chain α, ω-diols directly from alkanes in yeast. The rational pathway design and oxidation-pathway blocking highlight Y. lipolytica as a promising cell factory for alkane-based biomanufacturing and lay the groundwork for sustainable production of high-value diol precursors through targeted metabolic engineering.

## 1 Introduction

α, ω-Diols are essential building blocks for polyesters and polyurethanes (Gosecki et al., 2021; Ahsan et al., 2018), driving extensive research into microbial production via enzyme engineering, strain optimization, and cytochrome P450 (CYP450) monooxygenases (Ahsan et al., 2018; Lu et al., 2024; Lu et al., 2022; Kim et al., 2024; Lu et al., 2021). These compounds are classified by carbon chain length, with dramatically different production success rates. Short-chain diols (<C5) have achieved robust *de novo* synthesis from glucose, with *Clostridium beijerinckii* producing up to 26 g/L of 1,3-propanediol and engineered *E. coli* reaching 18 g/L of 1,4-butanediol (Fonseca et al., 2019; Yim et al., 2011) (Table 1). In contrast, mid-chain (C6–C12) and long-chain (>C12) diols remain orders of magnitude lower in production efficiency, with no *de novo* routes from simple carbon sources established.

**TABLE 1:** Engineered *Yarrowia lipolytica* strains constructed in this study.

Strain	Genotype	Description
YALI1	Po1g *ku70Δ*	Wild Type
YALI2	Po1g *ku70Δ mfe1Δ faa1Δ*	β-oxidation
YALI3	Po1g *ku70Δ mfe1Δ faa1Δ faldh4Δ*	Fatty aldehyde oxidation
YALI4	Po1g *ku70Δ mfe1Δ faa1Δ faldh1Δ faldh4Δ*	
YALI5	Po1g *ku70Δ mfe1Δ faa1Δ faldh1Δ faldh3Δ faldh4Δ*	
YALI6	Po1g *ku70Δ mfe1Δ faa1Δ faldh1-4Δ*	
YALI7	Po1g *ku70Δ mfe1Δ faa1Δ fao1Δ*	Fatty alcohol oxidation
YALI8	Po1g *ku70Δ mfe1Δ faa1Δ faldh1-4Δ fao1Δ*	Fatty alcohol dehydrogenation
YALI9	Po1g *ku70Δ mfe1Δ faa1Δ faldh1-4Δ fao1Δ fadhΔ*	
YALI10	Po1g *ku70Δ mfe1Δ faa1Δ faldh1-4Δ fao1Δ fadhΔ adh1Δ*	
YALI11	Po1g *ku70Δ mfe1Δ faa1Δ faldh1-4Δ fao1Δ fadhΔ adh1,6Δ*	
YALI12	Po1g *ku70Δ mfe1Δ faa1Δ faldh1-4Δ fao1Δ fadhΔ adh1,6,7Δ*	
YALI13	Po1g *ku70Δ mfe1Δ faa1Δ faldh1-4Δ fao1Δ fadhΔ adh1,6,7,8Δ*	
YALI14	Po1g *ku70Δ mfe1Δ faa1Δ faldh1-4Δ fao1Δ fadhΔ adh1,4,6,7,8Δ*	
YALI15	Po1g *ku70Δ mfe1Δ faa1Δ faldh1-4Δ fao1Δ fadhΔ adh1,3,4,6,7,8Δ*	
YALI16	Po1g *ku70Δ mfe1Δ faa1Δ faldh1-4Δ fao1Δ fadhΔ adh1,3,4,5,6,7,8Δ*	
YALI17	Po1g *ku70Δ mfe1Δ faa1Δ faldh1-4Δ fao1Δ fadhΔ adh1-8Δ*	

Current microbial synthesis of mid-to long-chain diols has been primarily demonstrated in bacteria such as *E. coli* and *Pseudomonas*, relying on hydroxylation of fatty acid derivatives or alcohols and achieving modest titers ranging from 79 to 1,400 mg/L (Ahsan et al., 2018; Fujii et al., 2006). The highest reported production is 1.4 g/L of 1,12-dodecanediol from 12-hydroxydodecanoic acid, while direct alkane conversion yields significantly lower titers, such as 108 mg/L of 1,8-octanediol from *n-*octane (Gudiminchi et al., 2012). This notable disparity in productivity between short-chain and mid/long-chain diols reflects fundamental bottlenecks in current approaches. The *E. coli*-based systems face critical limitations: heterologous CYP450 expression suffers from codon bias, protein misfolding, and complex electron transport requirements, while production remains restricted to expensive fatty acid feedstocks rather than abundant alkane substrates.

Alkanes, which are the most abundant hydrocarbons in petroleum and natural gas, represent an feedstock yet to be developed for diol production. Chemical alkane oxidation typically proceeds via harsh conditions that over-oxidize substrates to CO_2_, preventing selective hydroxylation and intermediate recovery (Munz and Strassner, 2015; Dubois, 2005). Biological systems offer superior selectivity through specialized alkane hydroxylases, including alkane-1-monooxygenases (AlkBs) and cytochrome P450s, which enable controlled oxidation to alcohols and subsequent derivatives (Guo et al., 2023; Williams et al., 2022; Funhoff et al., 2006; Scheller et al., 1998; Peters et al., 2003; Ayala and Torres, 2004). However, most alkane-utilizing bacteria require complex enzyme systems and cofactor regeneration, limiting their biotechnological application.

*Yarrowia lipolytica* emerges as an exceptional host for alkane bioconversion, naturally harboring 12 endogenous CYP52 family P450s (Alk1-12) alongside comprehensive alcohol oxidation machinery including 9 alcohol dehydrogenases (FADH, ADH), 1 fatty alcohol oxidase (FAO), and 4 fatty aldehyde dehydrogenases (FALDH) (Iwama et al., 2016; Takai et al., 2012; Gatter et al., 2014; Iwama et al., 2015; Iwama et al., 2014). Advanced synthetic biology tools for *Y. lipolytica* enable precise metabolic engineering (Larroude et al., 2018; Abdel-Mawgoud and Stephanopoulos, 2020; Wang et al., 2022; Abdel-Mawgoud et al., 2018), yet its potential for controlled diol production from alkanes remains largely unexplored.

**FIGURE 1:**
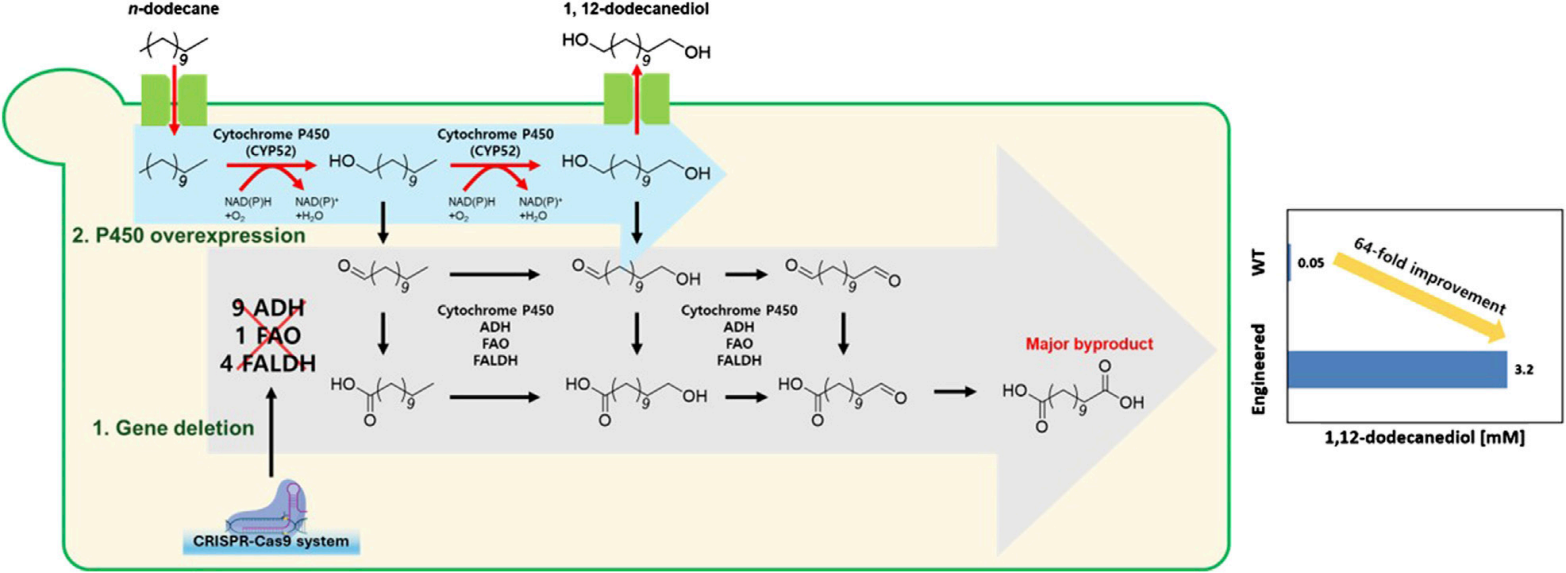
Metabolic engineering strategy for enhanced 1,12-dodecanediol production from *n*-dodecane in *Yarrowia lipolytica*.

Despite these biological capabilities, wild-type *Y. lipolytica* produces only 0.05 mM of 1,12-dodecanediol, reflecting inefficient flux control and competing oxidation pathways that convert valuable diol intermediates to terminal carboxylic acids. To overcome these limitations, we engineered *Y. lipolytica* using CRISPR-Cas9 to systematically block over-oxidation pathways (Supplementary Table S1) while overexpressing the Alk1 gene to enhance primary alkane hydroxylation capacity (Figure 1). Our optimized YALI17 strain achieved 1.45 mM 1,12-dodecanediol production, which is a 29-fold improvement over the wild type, and further pH optimization achieved 3.2 mM 1,12-dodecanediol production, demonstrating the first successful *de novo* production of mid-to long-chain α, ω-diols directly from alkanes in a yeast host. This breakthrough addresses the critical productivity gap in diol biotechnology, offering a sustainable route to valuable chemical intermediates from abundant hydrocarbon feedstocks while engineering the host’s native alkane metabolism to eliminate complex heterologous enzyme requirements.

## 2 Materials and methods

### 2.1 Strains, media and culture conditions

*E. coli* DH5α strains transformed with Alk repression vectors or Alk overexpression vectors were grown in Luria-Bertani (LB) medium (pH 7.0) supplemented with ampicillin (100 mg/L) and cultured at 37 °C. The *Y. lipolytica* strains were grown in YPD (20 g/L glucose, 20 g/L peptone, and 10 g/L yeast extract) medium (pH 6.5) or synthetic complete medium (20 g/L glucose, 6.7 g/L yeast nitrogen base without amino acids, supplemented with amino acid mix without leucine, pH 6.5) without L-leucine for 2 days and scale-up to the 20 mL of same medium in a 100 mL flask and incubated for another 2 days.

### 2.2 Construction of multiplexed P450 monooxygenase repression vectors

To construct vectors harboring a Cas9 gene, and multiple sgRNA scaffolds linked with the guiding sequence, the pCRISPRyl carrying a Cas9 protein and a sgRNA scaffold was employed as the cloning template. pCRISPRyl (Addgene #70007) was purchased from Addgene. For the simultaneous, combinatorial targeting of Alk genes in *Y. lipolytica*, another sgRNA scaffold sequence was inserted at the downstream of the original sgRNA scaffold site. Then, the guiding sequence (20 bp) for each Alk genes were inserted in the upper stream of each sgRNA scaffold sequence by overlapping PCR. PCR product was treated with DpnI for 16 h at 37 °C, transformed to *E. coli* DH5α and 6 colonies were picked and inoculated in LB medium supplemented with ampicillin (100 mg/L) and cultured at 37 °C. The next day, plasmids were purified for sequencing.

### 2.3 Construction of P450 ALK gene overexpression vectors

CYP450 alkane monooxygenase genes were PCR amplified from *Y. lipolytica* genome and cloned into pYl yeast expression vector using circular polymerase extension cloning (CPEC). pYl was constructed based on CRISPR vector pCRISPRyl (Schwartz et al., 2016) carrying a Cas9 protein and a sgRNA scaffold for use in *Y. lipolytica*. Since pCRISPRyl was a vector expressing cas9 protein and sgRNA in *Y. lipolytica*, we replaced the ORF of cas9 expression cassette with that of TA and removed the sgRNA scaffolds. To remove the sgRNA scaffolds from pCRISPRyl, two pairs of primers harboring SpeI or MfeI recognition sequence on the overhang was used to amplify Leu2 marker and the backbone of the vector (including AmpR marker, colE1 origin, Cas9 expression cassette, and CEN1-2). The amplified fragments were digested by SpeI and MfeI restriction enzymes and ligated by T4 DNA ligase. Finally, the intron sequence of the TEF promotor was amplified from *Y. lipolytica* genome and cloned at the 3′ of the TEF promoter by CPEC method to construct pYl.

### 2.4 Construction of over-oxidation deletion strains

To delete the genes, a CRISPR-Cas9 system for use in *Y. lipolytica* was introduced. Target-specific sequences (guiding sequences) targeting each gene were cloned at the 5′ end of the sgRNA scaffold in pCRISPRyl, forming sgRNAs that direct Cas9 to the gene of interest. For the donor DNA sequences to be stored and replicated at every use, donor DNA sequences were cloned in pUC-19 vectors by blue/white screening. In this study, we deleted the genes by removing around 500–1,000 bp long sequence in the middle of ORF regions. To achieve this, the donor DNA consisting of homologous arm of the upper and lower 1 kb sequence, respectively, of the deleted sequence was constructed. The assembled upper and lower fragments of the target gene were cloned into pUC19 vector by ligation. PCR amplified donor DNA and pCRISPRyl carrying a certain sgRNA were co-transformed into the *Y. lipolytica* strain and the transformed strains were grown on the SC-Leu agar plate for 2 days. After 2 days, colony PCR was performed by negative selection and the selected colonies were inoculated and grown in YPD medium for overnight. Next day, genomic DNA was extracted using Exgene Cell SV min kit (Geneall Biotech, Korea) and sequenced. Newly constructed strains were sub-cultured twice in YPD medium every 2 days to completely remove the remaining CRISPR plasmids in the cytosol. To construct a strain in which fatty acid degradation pathway is blocked, two genes were firstly chosen as deletion targets. Multifunctional enzyme1 (Mfe1, YALI0E15378g) is involved in the β-oxidation of fatty acids in peroxisome, and Long chain acyl-coA synthatase (Faa1, YALI0D17864g) oxidizes fatty acids for further β-oxidation. These two genes were sequentially deleted to give strain YALI2. Four fatty aldehyde dehydrogenases (YALI0F23793g, YALI0A17875g, YALI0E15400g, YALI0B01298g) were deleted in the listed order, followed by the deletion of fatty alcohol oxidase (YALI0B14014g) using the prescribed CRISPR-Cas9 strategy. Finally, 9 alcohol dehydrogenases (YALI0F09603g, YALI0D25630g, YALI0E17787g, YALI0A16379g, YALI0E15818g, YALI0D02167g, YALI0A15147g, YALI0E07766g, YALI0C12595g, YALI0B14014g) were sequentially deleted, as described in Table 1.

### 2.5 Biotransformation with resting cell reaction

For production of 1,12-dodecanediol, whole-cell biotransformation was performed by using dodecane as substrate. Cells grown in YPD or SC Leu-media were harvested and resuspended in the 100 mM potassium phosphate buffer (pH7.5), diluted to adjust their density to OD_600_ of 30. 100 mM Potassium phosphate buffer (pH 7.5) was supplemented with 2% (w/v) glucose, 50 mM *n-*dodecane, and OD_600_ = 30 cell. The initial reaction volume was 10–40 mL in baffled flask at 30 °C, 200 rpm. pH was adjusted to 7.5 with 5 N NaOH every 6 h of reaction. When constant pH control is needed, whole-cell biotransformation was conducted in a 916 Ti-Touch reactor (Metrohm, Switzerland) equipped with a magnetic stirrer and pH control. The reaction medium and conditions matched those used for shake flask experiments, with a total volume of 40 mL. Throughout the process, the pH was maintained at either 7.5 using the reactor’s pH controller and adjusted automatically with 2M NaOH.

### 2.6 Analysis of 1,12-dodecanediol and intermediates by gas chromatography

For GC analysis, samples were prepared by adding 1 mL of chloroform containing 1 mM of *n*-octanol as an internal standard to 200 μL to each sample. After vigorous vortexing for 1 min, samples were centrifugated at 13,000 rpm for 10 min. Next, 100 μL of the chloroform layer was separated, which was then mixed with 100 μL of N,O-Bis (trimethylsilyl) trifluoroacetamide and incubated at 50 °C in an oven for 30 min. Subsequently, the samples were transferred into GC vials for analysis. Gas chromatography-flame ionization (GC-FID) was conducted using an GC-2010 Plus instrument equipped with a HP-5 column (30 m × 0.32 μm × 0.15 μm), using helium as a carrier gas. The GC oven conditions were as follows: 90 °C (0.5 min), a 15 min ramp to 200 °C, a 5 min ramp to 280 °C, and held at 280 °C for 7 min. The split ratio was 20:1.

## 3 Results and discussion

### 3.1 Baseline alkane metabolism in *Y. lipolytica*

**FIGURE 2:**
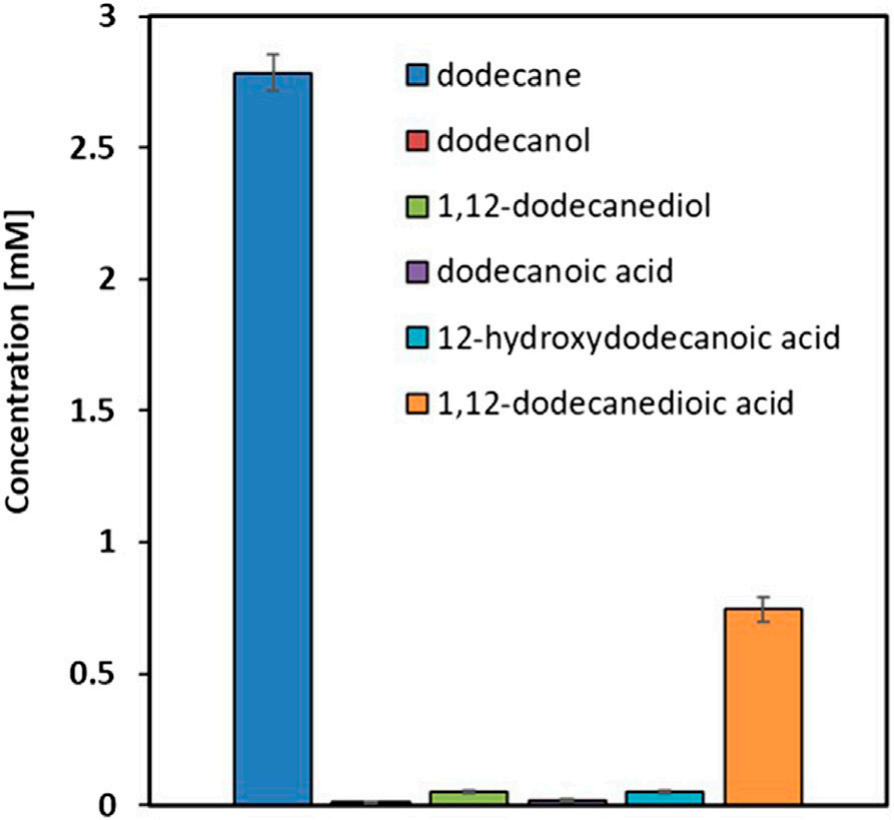
Comparison of *n-*dodecane and its oxidation products in *Y. lipolytica*YALI1 after 48 h of cultivation with 50 mM *n-*dodecane. Results are expressed as mean values ±standard deviation (n = 3).

The rational design of metabolically engineered strains requires comprehensive understanding of native metabolic flux distribution. We first characterized the baseline alkane metabolism in the control strain YALI1 to identify key bottlenecks limiting diol accumulation. When cultured for 48 h with 50 mM *n-*dodecane as substrate, YALI1 demonstrated characteristic terminal oxidation patterns (Figure 2). The metabolic profile revealed a predominant conversion of dodecane to 1,12-dodecanedioic acid (0.76 mM) as the major end product, with minimal accumulation of intermediates of interest (0.03 mM dodecanol, 0.05 mM 1,12-dodecanediol, and 0.04 mM 12-hydroxydodecanoic acid). This distribution confirms the efficiency of *Y. lipolytica’s* native over-oxidation machinery, where the 26 endogenous genes facilitate rapid conversion from alkanes to terminal carboxylic acids (Iwama et al., 2016; Takai et al., 2012; Gatter et al., 2014; Iwama et al., 2015; Iwama et al., 2014). The low diol-to-diacid ratio (0.06:1) demonstrates that intermediate metabolites are rapidly consumed by downstream oxidation pathways, particularly the 10 genes (FADH, ADH1-8, FAO1) responsible for fatty alcohol oxidation and 4 genes responsible for fatty aldehyde oxidation (FALDH1-4). However, after 48 h of cultivation, a substantial amount of *n-*dodecane substrate remained unconverted (2.8 mM), and the sum of the detected substrate and all measured metabolites fell far short of the 50 mM *n-*dodecane initially supplied. This indicates a clear gap in the mass balance. The YALI1 strain, derived from *Y. lipolytica* po1g, possesses only the ku70 gene deletion (to facilitate future gene deletion via homologous recombination), with all other metabolic pathways remaining intact. This genetic background allows very active alkane assimilation and consumption by the cells, likely resulting in the rapid uptake and potential routing of the substrate towards biomass formation or other unmeasured cellular metabolites, a phenomenon well-known for wild-type-like *Y. lipolytica* strains. This metabolic bottleneck analysis validated our engineering hypothesis: blocking these competing oxidation pathways should redirect metabolic flux toward diol accumulation rather than terminal oxidation to diacids.

### 3.2 Sequential gene deletion strategy and strain engineering

**FIGURE 3:**
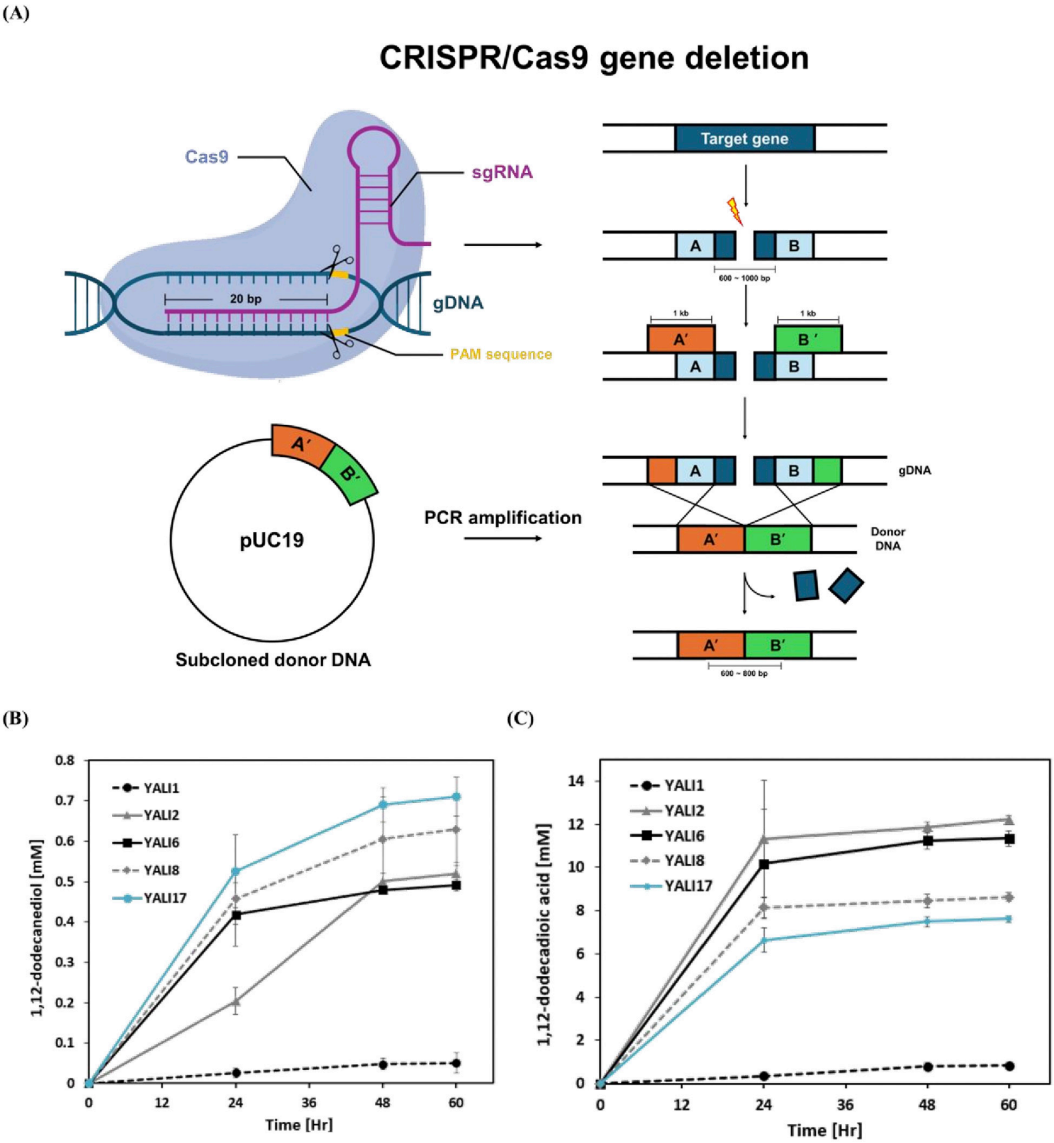
CRISPR/Cas9-mediated sequential gene deletions and their impact on alkane bioconversion in *Y. lipolytica*. **(A)** Schematic representation of CRISPR/Cas9 gene deletion strategy and homologous recombination for generating engineered strains. **(B)** Time-course production of 1,12-dodecanediol by parental and engineered YALI series strains cultivated with 50 mM *n-*dodecane. **(C)** Time-course production of 1,12-dodecanedioic acid by the same strains under identical conditions. Results are expressed as mean values ±standard deviation (n = 3).

To systematically disrupt over-oxidation pathways while maintaining alkane hydroxylation capacity, we constructed a series of deletion mutants using CRISPR-Cas9 technology (Figure 3A). The engineering strategy targeted three key metabolic nodes: β-oxidation (MFE1 deletion), fatty aldehyde oxidation (FALDH1-4 deletion), and fatty alcohol oxidation (FAO1, ADH1-9 deletion), along with deletion of FAA1, an acyl-CoA synthetase involved in fatty acid activation but not directly in the β-oxidation cycle (Table1). This sequential approach was designed to progressively eliminate oxidative pathways while preserving the primary CYP450-mediated alkane hydroxylation machinery essential for diol synthesis. The deletion progression from YALI2 through YALI17 created strains with increasingly compromised over-oxidation capacity, allowing for systematic evaluation of each pathway’s contribution to metabolic flux control. Time-course analysis of 1,12-dodecanediol production revealed distinct performance profiles among the engineered strains (Figure 3B). YALI17, containing the most comprehensive deletions, achieved the highest diol production from 50 mM *n-*dodecane (0.72 mM at 60 h), representing a 38% improvement over the control strain YALI2 (0.52 mM). Intermediate strains YALI6 and YALI8 showed progressive improvements (0.50 mM and 0.62 mM, respectively), confirming the cumulative effect of pathway blockade. The kinetic profiles demonstrated that diol accumulation continued throughout the cultivation period, suggesting successful flux redirection rather than mere metabolic disruption. This systematic engineering approach validated the metabolic control hypothesis while identifying YALI17 as the optimal strain configuration for enhanced diol production.

### 3.3 Enhanced diol production through metabolic pathway engineering

To systematically evaluate the effects of sequential gene deletions on metabolic flux partitioning, we compared time-course production profiles of 1,12-dodecanediol and 1,12-dodecanedioic acid among the parental strain and engineered *Y. lipolytica* derivatives (Figures 3B,C). The wild-type YALI1, containing only the ku70 deletion, maintained extremely low levels of both diol (0.05 mM) and diacid (0.82 mM), reflecting its native metabolic configuration where rapid β-oxidation and over-oxidation minimize intermediate accumulation. In contrast, YALI2 with targeted deletion of mfe1 and faa1 (key β-oxidation genes) consistently showed higher concentrations of all measured intermediates, including a marked increase in both diol (0.52 mM) and diacid (12.2 mM) pools. This result demonstrates that blocking β-oxidation efficiently preserves substrate-derived metabolites and supports sustained diol availability. Introduction of faldh1-4 deletions in YALI6 did not elevate diol titers (0.5 mM), while only slightly decreasing diacid formation (11.5 mM). Notably, additional deletion of fao1 in YALI8 resulted in a more pronounced diol increase (0.63 mM) and significant reduction of diacid (8.6 mM), indicating that FAO1 plays a central role in terminal oxidation. In the fully engineered strain YALI17, further removal of FADH and ADH1-8 yielded the highest diol levels (0.72 mM) and the lowest diacid accumulation (7.6 mM), but with only incremental improvement over YALI8. This finding suggests that deletion of FAO1 is consistently linked to a marked increase in diol titers and a substantial decrease in terminal oxidation-derived diacids, supporting its dominant role as the main fatty alcohol oxidase in *Y. lipolytica*. In contrast, removal of FALDH1-4 (responsible for fatty aldehyde oxidation) provides only modest effects on either diol or diacid levels, suggesting that FAO1-mediated over-oxidation is the rate-limiting step for intermediate loss rather than FALDH-dependent conversion (Gatter et al., 2014). Collectively, these data reveal that while broad suppression of over-oxidation machinery contributes to pathway redirection, targeted disruption of FAO1 is critical for maximizing diol retention. The modest further increase seen from additional ADH deletions indicates diminishing returns after the loss of FAO1, focusing future engineering efforts on this enzyme for effective flux redirection. This genetic hierarchy is clearly reflected in the time-course trends shown in Figures 3B,C.

The observed mass balance mismatch, in which the reduction in diacid production (4.6 mM) was not fully offset by the increase in diol (0.2 mM), likely reflects altered metabolite distributions stemming from the genotypical differences between YALI2 and YALI17, specifically the comprehensive deletion of genes such as FALDH1-4, FAO1, FADH, and ADH1-9. Previous studies have shown that such extensive genome engineering in *Y. lipolytica* leads to global metabolic pool remodeling and shifts in carbon flux, consistent with metabolite imbalances in engineered strains (Walker et al., 2021; Loira et al., 2012). Furthermore, the sustained diol production throughout the 60-h cultivation period in YALI17 indicates stable metabolic redirection without compromising cellular viability. The engineering approach successfully transformed *Y. lipolytica* from a strain optimized for complete alkane oxidation to one capable of controlled intermediate accumulation, achieving a 14-fold improvement in diol production (from 0.05 mM in YALI1 to 0.72 mM in YALI17). This represents a significant advancement in biotechnological diol production, demonstrating that systematic pathway engineering can overcome natural metabolic flux limitations in oleaginous yeasts for the production of valuable chemical intermediates from abundant hydrocarbon feedstocks.

### 3.4 Investigation of Alk genes as dual-function enzymes and repression strategy

**FIGURE 4:**
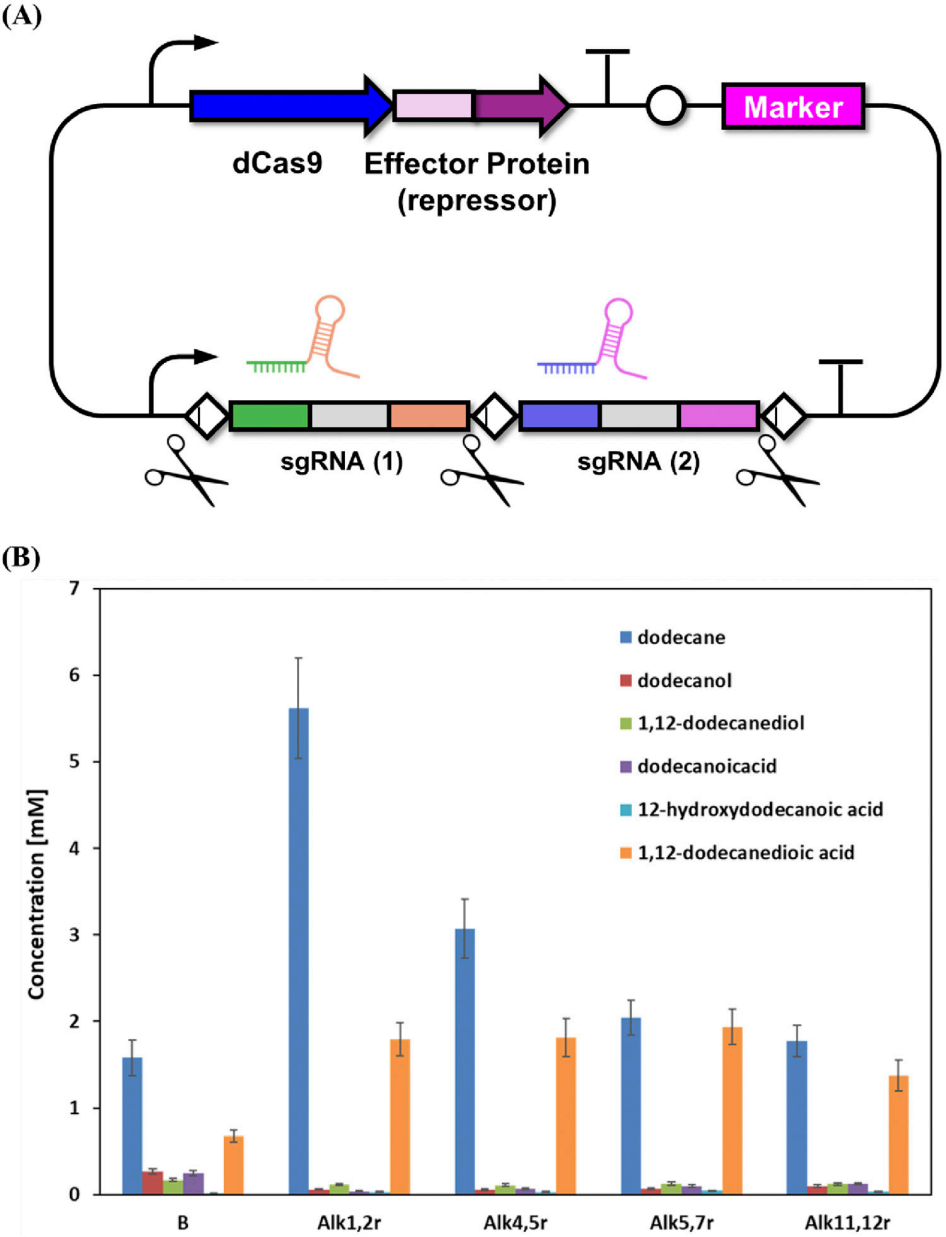
CRISPR/dCas9-based combinatorial repression of Alk gene clusters and its effect on alkane biotransformation in *Y. lipolytica*. **(A)** Schematic illustration of the multiplexed dCas9-sgRNA repression system targeting multiple Alk genes simultaneously. **(B)** Comparative analysis of *n-*dodecane and key oxidation product concentrations in control and Alk-repressed *Y. lipolytica* strains after whole-cell biotransformation. Results are expressed as mean values ±standard deviation (n = 3).

Despite systematic deletion of all predicted alcohol oxidation genes in the engineered strains, 1,12-dodecanedioic acid remained as the dominant product, indicating substantial residual oxidation activity in *Y. lipolytica*. Previous work has clearly established that Alk genes (CYP52 family) in *Y. lipolytica* function primarily as alkane terminal hydroxylases, catalyzing the initial step of converting alkanes to primary alcohols. The oxidation of fatty alcohol intermediates is instead mediated by distinct enzyme families, particularly fatty alcohol oxidase (FAO1) and multiple alcohol dehydrogenases (ADH1-7, FADH). There is, to date, no direct experimental evidence supporting a role for Alk enzymes in fatty alcohol oxidation. Based on these findings, our metabolic engineering strategy targeted suppression of undesired oxidative reactions, with the goal of enhancing diol accumulation and minimizing diacid production. This combinatorial approach was implemented using a CRISPR-based repression system, designed to systematically inhibit functionally redundant pathways and optimize carbon flux toward the target metabolite. The multiplexed repressor vector utilized dCas9-effector proteins with multiple sgRNAs to simultaneously repress Alk genes (Figure 4A). Alk genes were grouped for repression based on prior studies demonstrating distinct substrate specificities and functional overlap. For example, ALK1/ALK2 encode cytochromes essential for medium- and long-chain *n-*alkane assimilation, evidenced by their similar terminal hydroxylation activities (Iwama et al., 2016; Mauersberger et al., 2001; Fukuda, 2023). Likewise, ALK4/ALK5 show high ω-terminal fatty acid hydroxylation activity toward dodecanoic acid, while ALK5/ALK7 display overlapping substrate preferences in fatty acid and alkane assimilation. Lastly, ALK11/ALK12 represent CYP52 members with functionally limited substrate scopes, thus pairing them targets potential low-redundancy activities. These groupings are supported by systematic analysis of individual Alk paralogs and their substrate profiles in *Y. lipolytica*.

Comprehensive analysis of whole-cell biotransformation data demonstrates that combinatorial repression of Alk genes significantly altered substrate utilization and product distribution patterns, but not in the manner anticipated for enhanced diol biosynthesis (Figure 4B). All Alk-repressed strains including Alk1,2r, Alk4,5r, Alk5,7r, and Alk11,12r exhibited distinctly higher residual *n-*dodecane compared to the control (B), with concentrations ranging from 1.9 mM to 5.7 mM in repressed strains, versus only 1.6 mM in the control. This trend confirms that Alk gene repression efficiently dampened the upstream alkane hydroxylation step. Contrary to expectation, however, this reduction in *n-*dodecane consumption did not coincide with increased production of 1,12-dodecanediol or other key ω-oxidation intermediates. Across all repressed strains, the highest diol titer detected was only 0.12 mM (Alk11,12r), with most strains producing even less (0.03–0.09 mM), which is similar to or below the level in the control. Intermediate metabolites such as dodecanol and 12-hydroxydodecanoic acid also remained very low in all strains (0.04–0.07 mM and 0.02–0.07 mM, respectively), indicating constrained formation of downstream alcohols and hydroxyl acids following Alk repression.

Notably, all Alk-repressed strains showed a pronounced increase in terminal over-oxidation, as reflected by elevated 1,12-dodecanedioic acid levels (1.7–2.0 mM) compared to the control (0.6 mM). This apparent paradox may be explained by metabolic rerouting and compensation within the highly redundant P450 and ω-oxidation systems of *Y. lipolytica*. For example, it has been suggested that downregulation of one set of monooxygenases can lead to upregulation or increased activity of alternative oxidative enzymes, thereby maintaining or even accelerating terminal acid production despite diminished alkane consumption (Fickers et al., 2005; Hirakawa et al., 2009). Collectively, the data underscore that partial repression of select Alk gene clusters is insufficient to block over-oxidation and redirect metabolic flux to diol accumulation. Instead, by restricting substrate activation and intermediate formation without suppressing the entire ω-oxidation network, this strategy inadvertently favors terminal oxidation to dicarboxylic acids. This highlights the metabolic robustness and pathway redundancy in *Y. lipolytica*, indicating that more comprehensive targeting of the ω-oxidation machinery will be required to effectively modulate product selectivity.

### 3.5 Alk gene overexpression strategy and metabolic optimization

**FIGURE 5:**
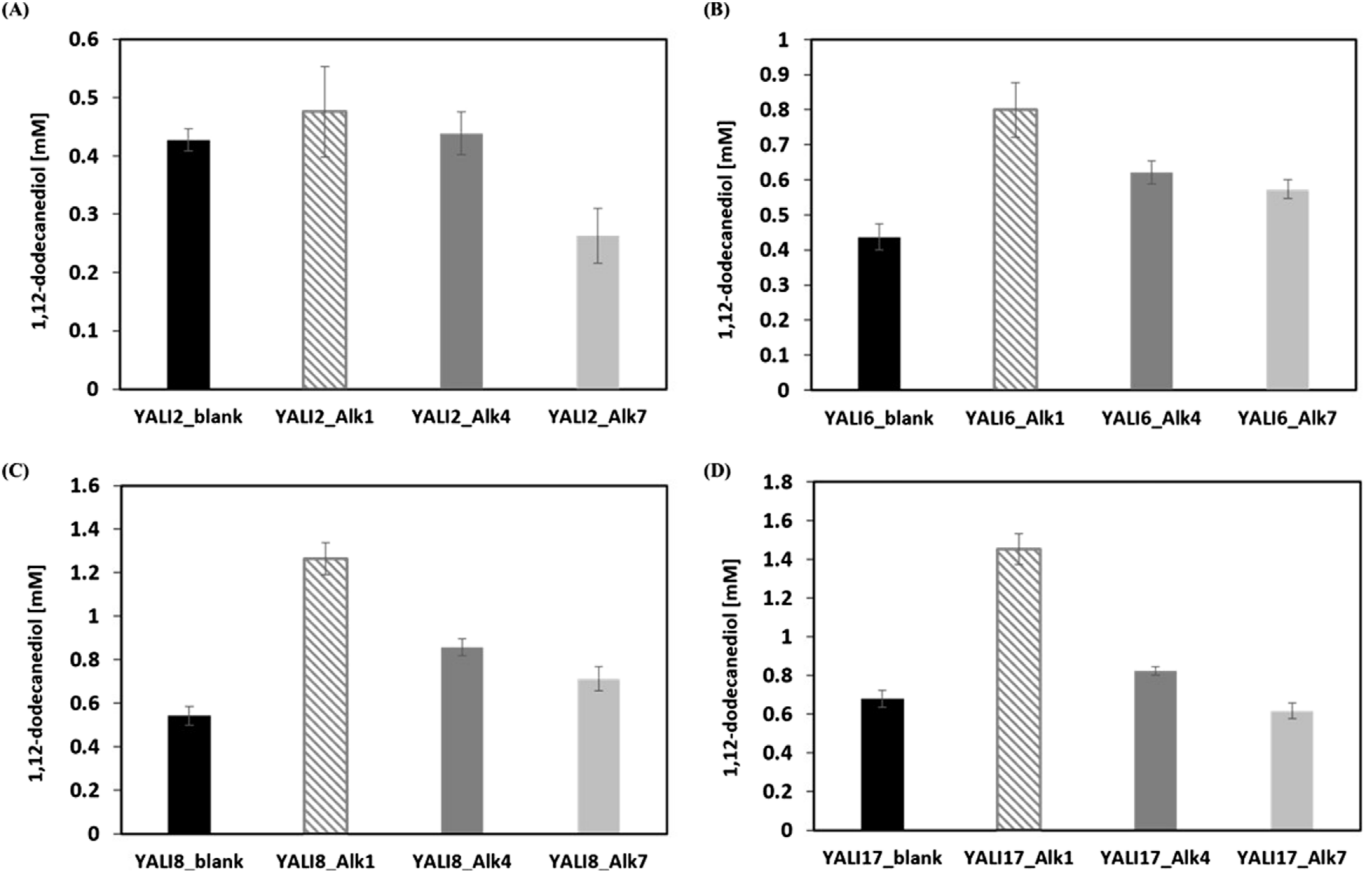
Effect of Alk gene overexpression on 1,12-dodecanediol production in engineered *Y. lipolytica* strains. **(A–D)** Quantification of 1,12-dodecanediol titers following overexpression of ALK1, ALK4, or ALK7 in deletion strain backgrounds YALI2 **(A)**, YALI6 **(B)**, YALI8 **(C)**, and YALI17 **(D)**, compared to respective empty vector controls (blank). Results are expressed as mean values ±standard deviation (n = 3).

Based on the failure of the repression approach, we focused on an overexpression strategy predicated on the hypothesis that alkane hydroxylation, rather than over-oxidation, was the rate-limiting step in diol production. 3 selected Alk genes (ALK1, ALK4, ALK7) from each repression pair were cloned into the yeast expression vector (pYl) harboring a TEF_intron_ promoter and a yeast replication origin and introduced into the engineered deletion strains YALI2, YALI6, YALI8, and YALI17. To address the functional contribution of different Alk family members, Alk1, Alk4, and Alk7 were selected for overexpression based on their genetic relationship (one from each repression pair), previous reports of monooxygenase activity, and their genomic abundance in *Y. lipolytica*. Alk1 was chosen as the principal representative due to its confirmed activity toward medium-chain alkanes in several studies. Alk4 and Alk7, despite showing negligible activity toward *n*-alkanes under standard conditions (Iwama et al., 2016), were included to evaluate whether pathway deletions could reveal context-dependent increases in substrate hydroxylation or alter their catalytic profile. This approach allowed us to rigorously compare the alkane transformation potential of multiple Alk paralogs in engineered metabolic backgrounds and to test if uncharacterized or latent activities could be functionally relevant for diol production in *Y. lipolytica*. Whole-cell biotransformation experiments employing 50 mM *n-*dodecane confirmed significant enhancement of 1,12-dodecanediol production by targeted overexpression of Alk genes, with specific effects clearly visualized in Figures 5A–D. Across all strain backgrounds, the empty vector controls (YALI2_blank, YALI6_blank, YALI8_blank, YALI17_blank) achieved diol titers very similar to, or slightly lower than their corresponding parental strains without the empty vector (for example, YALI2_blank: 0.43 mM vs. YALI2: 0.5 mM; YALI17_blank: 0.68 mM vs. YALI17: 0.72 mM). This minor reduction is likely attributed to a moderate metabolic burden imposed by maintenance and expression of the vector backbone, which may slightly reduce the maximum attainable flux for diol biosynthesis due to resource allocation or possible basal level expression effects.

A notable pattern was observed when Alk genes were overexpressed in increasingly engineered genetic backgrounds. The positive impact of Alk1 overexpression became more pronounced as additional competing oxidative pathways were deleted. In YALI2_Alk1, diol titer was 0.47 mM, which was only marginally higher than the control, whereas in YALI17_Alk1, diol production surged to approximately 1.45 mM, outstripping the blank control by more than two-fold (YALI17_blank: 0.68 mM). A similar but stepwise trend was observed for Alk4 and Alk7: while their overexpression in YALI2 only raised diol titers modestly (YALI2_Alk4: 0.44 mM; YALI2_Alk7: 0.30 mM), the increases became progressively more substantial in YALI6, YALI8, and most dramatically in YALI17 (YALI17_Alk4: 0.83 mM; YALI17_Alk7: 0.61 mM). This synergistic effect indicates that removing metabolic sinks via multi-gene knockout substantially allows improved substrate channeling to the Alk-overexpressed step and maximizing diol accumulation.

Among all genes tested, Alk1 overexpression consistently resulted in the highest 1,12-dodecanediol titers in every strain background. For instance, YALI6_Alk1 produced 0.80 mM and YALI8_Alk1 produced 1.26 mM. This superior performance aligns with previous literature identifying Alk1 as the principal monooxygenase for mid-chain alkane assimilation in *Y. lipolytica*, likely due to its catalytic efficiency and substrate specificity for C12 alkane substrates. Despite these improvements, a substantial amount of diacid (typically >10 mM) continued to accumulate across all conditions, highlighting the persistent robustness of terminal oxidation in *Y. lipolytica*. This outcome highlights both the capability and limitation of this engineering approach: while enhanced Alk expression in the context of maximal pathway pruning can substantially elevate diol yields (achieving up to 1.45 mM diol in YALI17_Alk1), it remains challenging to completely divert flux away from diacid formation. Overall, these results establish the effectiveness of Alk1 overexpression in combination with systematic pathway deletions for maximizing diol production, while providing a clear rationale for further interventions to reduce residual terminal oxidation and improve product selectivity.

### 3.6 Impact of pH control strategy on 1,12-dodecanediol production

The pH of the reaction environment plays a crucial role in influencing enzymatic activity, both *in vitro* and *in vivo*. In whole-cell biocatalytic systems where high substrate concentrations are used, maintaining optimal pH can be challenging, especially when pH adjustments are made manually. In this context, it was observed that the pH of the reaction mixture would fall to around 5.3–6.5 before each scheduled adjustment, after which 5M NaOH was added to restore it. This fluctuation indicates the limitations of manual pH regulation. Consequently, using a pH-stat device to continuously maintain the desired pH offers significant practical advantages, ensuring more stable reaction conditions and potentially improving product yields.

**FIGURE 6:**
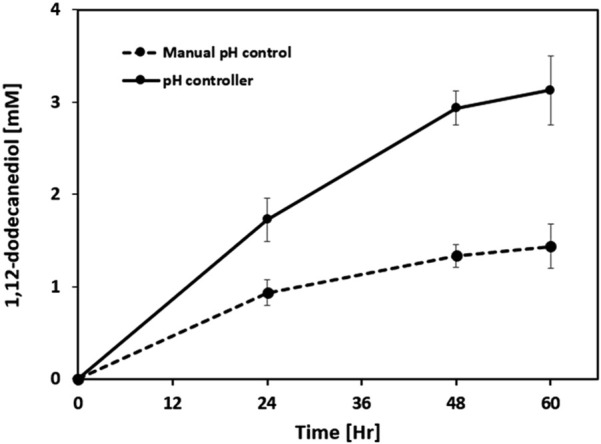
Effect of pH control strategy on 1,12-dodecanediol production in whole-cell biotransformation. Comparison of time-course 1,12-dodecanediol titers between manual pH adjustment (every 6 h) and continuous pH control at 7.5 using a pH controller in a 40 mL bioreactor. Results are expressed as mean values ±standard deviation (n = 3).

For ω-hydroxylation of dodecanedioic acid, alkaline conditions are reported to be beneficial for both reaction rate and regioselectivity (S et al., 2012). To assess the impact of pH control strategy, 1,12-dodecanediol production was compared between manual and automated (pH controller) adjustment at pH 7.5 in a 40 mL reactor (Figure 6). Although no direct measurements of Alk enzyme activity in *Y. lipolytica* at pH 7.5 have been previously reported, mechanistic analogy can be drawn from studies on CYP52 monooxygenases in *Candida bombicola*, where optimal fatty acid hydroxylation occurs under mildly alkaline conditions (pH 7.5–8.0) (Huang et al., 2014). This supports the rationale for selecting pH 7.5 in our biotransformation to maximize ω-hydroxylation efficiency via Alk enzymes in *Yarrowia lipolytica*.

In the manual pH control group, pH was allowed to drift down and then restored every 6 h, whereas the pH controller maintained pH constantly at 7.5. After 60 h, the titer of 1,12-dodecanediol reached approximately 1.4 mM under manual control and 3.2 mM under automated pH control. The production rate in the pH controller group was more than double that of the manual group, demonstrating a clear beneficial effect of constant alkaline pH. These results reinforce previous reports that mildly alkaline pH promotes ω-hydroxylation activity of fatty acid-transforming enzymes. Intermittent manual control allowed the pH to drop to suboptimal acidic values, limiting enzyme efficiency and product yield. In contrast, maintaining a constant alkaline pH (7.5) using a pH controller facilitated higher diol titers and improved bioconversion efficiency. The findings underscore the importance of tight pH regulation for the practical application and scale-up of whole-cell ω-hydroxylation reactions in bioreactor settings.

## 4 Conclusion

This study successfully establishes *Yarrowia lipolytica* Po1g strain as a microbial platform for producing medium-to long-chain α, ω-diols directly from alkanes, marking a significant advancement in biomanufacturing. By deleting 10 genes responsible for fatty alcohol oxidation and 4 for fatty aldehyde oxidation, the engineered strain YALI17 achieved a 29-fold increase in 1,12-dodecanediol production, from 0.05 mM (1.01 mg/L) in wild type to 1.45 mM (293 mg/L) with Alk1 overexpression, starting from 50 mM (8.52 g/L) n-dodecane substrate. This corresponds to a molar yield of 2.9% and a mass yield of approximately 3.4% based on substrate conversion. Further enhancement in pH controller trials increased titer to 3.2 mM (647 mg/L), more than doubling flask production under the same substrate conditions. However, a substantial accumulation of 1,12-dodecanedioic acid remains, indicating the presence of residual or alternative oxidation pathways that require further characterization. Repressing or knocking out the entire ALK1-12 gene family may offer a strategy to further reduce by-product formation and improve diol yield by limiting over-oxidation. Future work will focus on further characterizing Alk enzyme substrate specificities, applying protein engineering to reduce unwanted oxidation, and identifying enzymes contributing to diacid by-product formation. Additionally, engineering strategies to enhance alkane uptake, such as overexpressing or mutating ABC transporters ABC2 and ABC3, will be explored to improve substrate assimilation and tolerance. Evaluation of strain scalability and bioreactor optimization will also remain priorities. Expanding substrate and product scope through continued metabolic engineering and integrating these processes into sustainable biomanufacturing platforms will be essential for advancing microbial alkane valorization. Overall, this work lays a strong foundation for alkane valorization using *Y. lipolytica* and highlights both its potential and the challenges to be addressed in advancing sustainable microbial production of valuable diols.

## Data Availability

The original contributions presented in the study are included in the article/Supplementary Material, further inquiries can be directed to the corresponding author.
