# Supplementary material for: Metabolic engineering of Yarrowia lipolytica for enhanced microbial production of medium-chain α, ω-diols from alkanes via CRISPR-Cas9 mediated pathway optimization and P450 alkane monooxygenase overexpression
Source: Front Bioeng Biotechnol. 2025 Oct 23;13:1695661. doi: 10.3389/fbioe.2025.1695661 (PMC12588895; doi:10.3389/fbioe.2025.1695661)
Supplement: Supplementary file 1 [file Table1.docx]

Supplementary Material

# Supplementary Figures and Tables

## Table S1. Target genes in *Y. lipolytica* for deletion.

| **Enzyme** | **Gene** | **Name** | **Amino acids** |
| --- | --- | --- | --- |
| β-oxidation-related | YALI0E15378g | MFE1 | 901 |
|  | YALI0D17864g | FAA1 | 691 |
| Fatty-alcohol oxidase | YALI0F09603g | FADH | 381 |
|  | YALI0D25630g | ADH1 | 349 |
|  | YALI0E17787g | ADH2 | 351 |
|  | YALI0A16379g | ADH3 | 349 |
|  | YALI0E15818g | ADH4 | 492 |
|  | YALI0D02167g | ADH5 | 346 |
|  | YALI0A15147g | ADH6 | 348 |
|  | YALI0E07766g | ADH7 | 346 |
|  | YALI0C12595g | ADH8 | 424 |
|  | YALI0B14014g | FAO1 | 609 |
| Fatty-aldehyde dehydrogenase | YALI0A17875g | FALDH1 | 534 |
|  | YALI0E15400g | FALDH2 | 522 |
|  | YALI0B01298g | FALDH3 | 530 |
|  | YALI0F23793g | FALDH4 | 520 |
